# The Optimal Duration of Adjuvant Chemotherapy in Colon Cancer

**DOI:** 10.3390/cancers12092509

**Published:** 2020-09-03

**Authors:** Maike Collienne, Dirk Arnold

**Affiliations:** Department of Oncology and Hematology, Asklepios Klinik Altona, Asklepios Tumorzentrum Hamburg, 22763 Hamburg, Germany; m.collienne@asklepios.com

**Keywords:** colon cancer, adjuvant therapy, peripheral sensory neurotoxicity

## Abstract

**Simple Summary:**

This review provides an overview of adjuvant chemotherapy in colon cancer over the last 30 years. Key questions address choice of regimen, treatment duration, treatment-dependent toxicity and possible prognostic/predictive factors. Thus, main focus is on the International Duration Evaluation of Adjuvant therapy (IDEA) collaboration which was founded to investigate whether a shortened (3 months) of adjuvant therapy containing a fluoropyrimidine and oxaliplatin is comparable to the current/former standard of care (duration of 6 months). Results of this collaboration will be discussed and evaluated in the context of achieving the best efficacy possible while reducing severe toxicities, and future prognostic/predictive factors.

**Abstract:**

Adjuvant chemotherapy for colon cancer (UICC stage II and III) has been under investigation over the last 30 years, regarding treatment duration and regimens. In this review, choice of regimen, its duration, possible limitations and future perspectives are discussed. Monotherapy with 5-fluorouracil was followed by addition of oxaliplatin, resulting in improved 3-yr disease free survival (DFS) and overall survival (OS) rates, but also increased peripheral sensory neurotoxicity (PSN). The International Duration Evaluation of Adjuvant therapy (IDEA) collaboration demonstrated less toxicity, especially PSN, when shortening treatment duration to 3 months. However, formally, the anticipated non-inferiority of 3 months with fluoropyrimidine (FP)/oxaliplatin over 6 months (at 3-yr DFS) was not met for all patients groups, although subgroup analyses showed non-inferiority with capecitabine/oxaliplatin (CAPOX) rather than with FOLFOX, and also in relation to the prognostic information (e.g., clinical low-risk group, pT1-3 N0). In addition, first data of newer parameters like Immunoscore^®^ and ctDNA show promising results as stratification parameters. Further investigations to better define clinical risk groups and prognostic factors are mandatory. Besides this, individual decision-making of treatment intensity (FP or FP/oxaliplatin) and duration should always consider patient characteristics and preferences, also given the absolute relatively small differences and their clinical relevance.

## 1. Introduction

**Figure 1:**
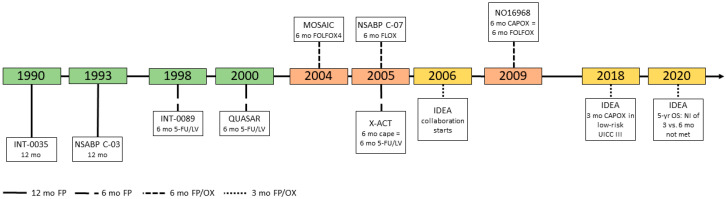
Years of adjuvant chemotherapy in colon cancer: from 5-FU (backbone) to FP/oxaliplatin (5-FU 5-fluorouracil, FP fluoropyrimidine, LV leucovorin, FOLFOX4 5-FU/LV + oxaliplatin, FLOX FULV + oxaliplatin, cape capecitabine, CAPOX capecitabine + oxaliplatin, NI non-inferiority, OX oxaliplatin).

Over the last three decades, adjuvant chemotherapy for stage III colon cancer has been adjusted in terms of *treatment intensity*, by chosen regimens, and of *treatment duration*, since efficacy—and toxicity—have been seen as a composite variable of both factors (Figure 1). Therefore, modifications of both factors are contributing to the choice of the best treatment option for a respective situation.

## 2. Fluoropyrimidines as Single Agent

Historically, at the end of the 20th century, adjuvant chemotherapy containing a 5-fluorouracil (5-FU) backbone alone or with “biomodulation”, e.g., by the anthelmintic drug levamisole (LEV), was seen as standard of care, resulting in prolonged disease-free survival and overall survival rates compared to clinical observation or each compound alone [1,2]. However, treatment duration of all compounds was not less than 12 months. In the following years, several studies investigated variations of regimens concerning treatment duration, possible combinations and doses of 5-fluorouracil (5-FU), leucovorin (LV), and levamisole (LEV) [3]. Notably, the first trial investigating a treatment intensification associated with a shortened duration was Intergroup-0089 trial [4] which showed no differences in DFS and OS rate between 6 and 12 months of adjuvant therapy consisting of four different regimens (5-FU + low-dose LV (LDLV), 5-FU + high-dose LV (HDLV), LDLV + LEV, and 5-FU + LEV): The latter did not provide any benefit in comparison to LDLV and HDLV, and there was no difference between 6 to 8 and 12 months of LDLV and HDLV. In consequence, treatment duration was shortened to 6 months.

Moreover, the QUASAR trial showed no benefit of survival adding levamisole to 5-FU/LV, and administering high or low doses of LV [5]. In respect of these results, the standard regimen for adjuvant therapy was revised to 5-FU/LV with a duration of six months. Also, in two large phase III trials comparing oral fluoropyrimidines (capecitabine and tegafur/folinic acid (UFT/FA)) to standard 5-FU/LV, a modification of treatment duration was not examined. Thus, the 6 months period was consolidated as standard of care for any fluoropyrimidine as single agent [6,7,8,9,10].

## 3. The Addition of Oxaliplatin

While adhering to a period of six months, regimens were proven to be superior when adding oxaliplatin: In 2004, results of the Multicenter International Study of Oxaliplatin/ Fluorouracil/Leucovorin in the Adjuvant Treatment of Colon Cancer (MOSAIC) demonstrated a significant improvement of the 3-year DFS in patients treated with a doublet regimen (oxaliplatin with 5-FU/LV [FOLFOX4]) to monotherapy (5-FU/LV) regarding the all-patient cohort (HR 0.77, 95% CI, 0.65 to 0.91) as well as the subgroup UICC III (HR 0.76, 95% CI, 0.62 to 0.92). Similar results were reported by two other phase III trials confirming a higher disease-free survival rate at three years by adding oxaliplatin: the NSABP C-07 trial compared a bolus 5-FU/LV regimen with oxaliplatin (FLOX) with 5-FU/LV and the NO 16968 trial investigated CAPOX and 5-FU/LV. In long-term, these results were confirmed by their final analyses, indicating also a significant improvement of overall survival (MOSAIC trial and NO 16968 trial with statistical significance for UICC III patients) [11,12,13,14]. 

The addition of oxaliplatin to the various FP backbone regimen over the treatment period of 6 months in the above mentioned various regimen resulted in relative high cumulative doses of oxaliplatin: 765, 1020 and 1040 mg/m^2^ referring to NSABP C-07, MOSAIC, and NO 16968, respectively. As a result of that, peripheral sensory neuropathy (PSN) occurred more often in doublet regimens: approximately 10% of patients developed grade 3/4 PSN as it is a well-known cumulative, dose-depended side effect of oxaliplatin. The median time between first treatment and onset of PSN (grade 2 to 4) was 60 days (range, 1 to 213 days) in the NO 16968 trial [15]. The incidence of PSN decreased over time: 20.9% (FLOX) and 3.9% (FULV) of patients (NSABP C-07) [16] had PSN (grade 2 or 3) after one month and 18 months, respectively. PSN resolved earlier using CAPOX (median duration 35 days (range, 1 to 621 days) [17] than FLOX (median duration 9.0 months) [16].

The aim of further trials was to achieve the best efficacy of adjuvant therapy possible while shortening its duration due to cumulative toxicity. To finally investigate the optimal duration of adjuvant chemotherapy in stage III disease, the International Duration Evaluation of Adjuvant therapy (IDEA) Collaboration was founded in 2006 [18].

## 4. IDEA Collaboration: Limiting Cumulative Doses of Oxaliplatin Whilst Maintaining Benefit

The goal to investigate whether three months of adjuvant therapy with a fluoropyrimidine (FP)/ oxaliplatin combination compared to a treatment duration of 6 months would result in a markedly reduced toxicity without clinically meaningful loss of efficacy arose in several scientific groups. Therefore, an international project including six multicenter, randomized phase III trials conducted in 12 countries: Three or Six Colon Adjuvant (TOSCA) trial (Italy) [19,20], Short Course Oncology Treatment (SCOT) trial (UK) [21], IDEA France trial [22], intergroup Cancer and Leukemia Group B/Southwest Oncology Group (CALGB/SWOG) trial 80702 (US), Hellenic Oncology Research Group (HORG) trial (Greece) [23], and Adjuvant Chemotherapy for colon cancer with HIghEVidencE (ACHIEVE) trial (Japan) [24,25] was initiated, aiming a preplanned combined analysis.

Primary hypothesis of the IDEA trial was to evaluate whether 3 months of adjuvant therapy with FP/oxaliplatin were non-inferior to a duration of 6 months. Non-inferiority was technically defined as achieved if the upper limit of the two-sided 95% confidence interval (CI) of the Hazard Ratio (HR) for DFS would be less than 1.12. This 12% reduction of efficacy which would have been accepted as being non-inferior was calculated from the initial findings of the MOSAIC trial, resulting in a 24% (relative) reduction of DFS with 6 months of 5-FU/LV/oxaliplatin (in FOLFOX4) were compared to no oxaliplatin (LV5FU2 regimen), in a ‘linear’ logical assumption: If 6 months oxaliplatin reduce the risk for relapse by 24%, then 3 months should prompt into a 12% reduction. To provide a power of 90%, a preplanned sample size of 10,500 patients was assumed over all trials and at least 1000 patients in every single trial [26].

Prerequisites for the collaboration were a prospective trial design aiming to investigate the most advantageous period of adjuvant therapy in stage III colon cancer, randomization of three- and six-months groups administering FP/oxaliplatin and 3-year DFS as the primary endpoint to (statistically) exclude a non-inferiority. Besides this, the patient-relevant gain in reduced toxicity was another important endpoint.

In addition, all trials were unblinded to physicians and patients, and treatment regimens (FOLFOX or CAPOX) were depended on physician’s choice. Statistical analyses were conducted in a modified intention-to-treat (mITT) population, which consists of all patients being randomized and having received at least one dose of chemotherapy. Preplanned analyses were conducted by tumor stage, nodal status, and treatment. Unplanned post-hoc analyses were performed defining (prognostic) low- and high-risk groups.

A total of 12,834 patients with stage III colon cancer were enrolled in the aforementioned trials and included in the main analysis of the IDEA collaboration. Since the choice of FP was investigator’s decision, 60% of patients received FOLFOX regimen, which was most often provided in the IDEA France (90%) and the CALGB/SWOG 80702 trial (100%). The distribution of CAPOX regimen among the six trials varied from 0% (CALGB/SWOG 80702) to 75.1% of patients (ACHIEVE) [18]. For instance, 66.5% and 58.2% of patients were treated with CAPOX in the SCOT and HORG trial, respectively.

With respect to the primary endpoint, IDEA failed to show a statistical non-inferiority of 3 months over 6 months of adjuvant therapy within the defined margins, since an overall 7% reduction in efficacy (HR 1.07) was also associated with an upper 95% CI limit crossing the pre-specified margin of 1.12 (95% CI 1.00 to 1.15). Somehow correlating to the 7% decrease in efficacy, 3-yr DFS rates in the 3- and 6-months group were 74.6% and 75.5%, respectively.

In terms of patient benefit, treatment dependent toxicities were significantly reduced: acute (during treatment or within one month thereafter) peripheral sensory neurotoxicity (≥grade 2) was significantly less often observed after a duration of three months of treatment independent on chosen regimen: 16.6% vs. 47.7% of the FOLFOX group and 14.2% vs. 44.9% of the CAPOX group had PSN grade 2 to 4 [18]. Long-term results of the ARCHIEVE trial confirmed these findings: persistent PSN within three years after last chemotherapy dose was significantly less often observed in patients having received three months of chemotherapy (8% vs. 21% for CAPOX (*p* < 0.001) and 16% vs. 34% for FOLFOX (*p* < 0.001)) [25]. Likewise, diarrhea, bone marrow depression, nausea, mucositis, and fatigue were less frequently seen. Subgroup analyses, however, provided several relevant findings, discussed in the following sections. 

### 4.1. Role of Chemotherapy Regimen

In the patient cohort treated with capecitabine plus oxaliplatin (CAPOX regimen), the non-inferiority criteria of 3 months of adjuvant therapy was statistically met (HR 0.95, 95% CI, 0.85 to 1.06), with a corresponding 3-yr DFS rate of 75.9% and 74.8% in the 3- and 6-months groups, respectively.

In the all-patient cohort, the shorter treatment period with FOLFOX was associated with existence of inferiority (HR 1.16, 95% CI, 1.06 to 1.26). 3-yr DFS rates were 76.0% and 73.6%, respectively (Table 1).

Besides this, the efficacy of both chemotherapy regimens—independent of their duration—cannot be compared in this project due to its design and the unequal distribution within the six different trials.

### 4.2. Role of Clinical Prognostic Factors: T- and N-stage

Subgroup analyses depending on tumor stage and nodal status showed poorer outcomes in patients with some unfavorable prognostic criteria. E.g., patients with T4 stage treated for 3 months had a poorer outcome (HR 1.16,95 % CI, 1.03 to 1.31), whereas there was no impact on nodal status (N1 or N2) alone (N1: HR 1.07, 95% CI, 0.97 to 1.17; N2: HR 1.07, 95% CI, 0.96 to 1.19).

Regarding the (unplanned) subgroup analysis of clusters of (so defined) *low-risk* (T1-3 N1) and *high-risk* (T4 and/or N2) criteria, those patients fulfilling high-risk criteria benefit from a longer duration of adjuvant therapy (HR 1.12, 95% CI 1.03 to 1.23) with a 3-yr DFS in 3 and 6 months of 62.7% and 64.4%, respectively. In the low-risk group, criteria of non-inferiority of the shorter treatment period were met (HR 1.01, 95% CI 0.90 to 1.12). 

### 4.3. Combining Clinical Risk Factors and Chemotherapy Regimen

Combining the *low-risk* (T1-3 N1) situation and ‘chosen treatment’ (choice of FP) as parameters, a limitation of the CAPOX regimen—with a duration of 3 months—is statistically non-inferior to 6 months treatment duration (HR 0.85, 95% CI, 0.71 to 1.01). By contrast, with FOLFOX; the absolute difference of 3-yr DFS of the *low-risk* group amounts 1.6%; however, non-inferiority was formally not proven (HR 1.10, 95% CI, 0.96 to 1.26). Likewise, although the (absolute) differences are small (0.1%, corresponding to a HR 1.02), the non-inferiority of the shorter duration could not be demonstrated for the combination *high-risk* and CAPOX (3 months) (95% CI, 0.89 to 1.17). The only combination of parameters with a clear inferiority for a 3 months treatment period was of FOLFOX (3 months) used in the *high-risk* group (HR 1.20, 95% CI, 1.07 to 1.35) (Table 1).

### 4.4. Newer Parameters: Immunoscore and cfDNA

Immunoscore^®^ (IS) is a commercially available assay incorporating various immunogenic factors, e.g., density of CD3+- and CD8+ T-cells in tumors and their invasive margins [28,29,30]. The score is based on immune-histochemical findings in correlation with digital analyses. High levels of IS were previously found to be correlated with a lower risk of recurrence [30]. Within the IDEA France trial, the pre-specified subgroup analyses included IS, limited to the patient cohort treated with FOLFOX (and not those being treated with CAPOX). Like in the prior international, multicenter validation trial for IS, DFS was significantly longer in patients harboring tumors with intermediate and high levels of IS. Interestingly, *low-risk* patients (T1-3 N1) with intermediate and high IS had a significant longer DFS when treated for 6 rather than for 3 months. In contrast, (clinical) *high-risk* patients (T4 or N2) with low IS did not benefit from a prolonged period of adjuvant therapy. In those patients, it seems to be crucial to identify other risk factors to determine optimal duration of adjuvant therapy.

Moreover, cell-free tumor DNA (ctDNA) was first investigated in a prospective trial for early colon cancer by the IDEA France trial. 805 patients were included in the post hoc analysis: 13.5% were ctDNA positive after surgery, which was most often associated with high-risk criteria (G3 and/or tumor perforation). 2-yr DFS was significant shorter in ctDNA-positive patients. In a multivariate analysis, ctDNA was an independent prognostic factor. However, adjuvant treatment for six months was superior to 3 months for both ctDNA-negative and -positive patients [31].

In combination of these results of the IDEA France trial and the main analysis of the IDEA collaboration, identifying *low-risk* patients who clearly benefit from shorter treatment periods is not as simple as suggested in subgroup analyses. Further trials to evaluate the efficacy of CAPOX vs. FOLFOX in correlation with Immunoscore^®^, ctDNA or other potential predictive factors are needed.

#### 4.4.1. IDEA: Long-Term Results

Recently, final results of IDEA were presented at the 56th American Society of Clinical Oncology (ASCO) annual meeting. In accordance to the assumptions of non-inferiority of the shorter duration at 3-yr DFS, the non-inferiority margin of 5-yr OS was also referred to the results of the MOSAIC trial: HR was defined as achieved if the upper limit of the two-sided 95% CI of the HR would be less than 1.11 regarding the absolute OS gain of 22% administering doublet therapy in the MOSAIC trial. Results show that non-inferiority was again statistically not met in OS at 5 years (HR 1.02, 95% CI 0.95 to 1.11), even though the absolute difference of OS rate among the groups (82.4% vs. 82.8%, 3 vs. 6 months, respectively) was neglectable (0.4%) [32]. In respect to the chosen regimen and risk group a shorter duration of CAPOX met the criteria of non-inferiority in the low-risk group (HR 0.85, 95% CI, 0.69 to 1.04). Patients classified as high-risk benefited from a duration of six months administering FOLFOX (HR 1.12, 95% CI, 0.98 to 1.27). These results confirm the 3 yr DFS rates.

While transferring these final results to clinical practice different aspects should be considered: The aforementioned statistical analyses including the definition of non-inferiority based on the results of a former clinical trial (MOSAIC) with its statistical hypotheses and limits. The IDEA collaboration exists of six different trials which were not designed at the same time and, therefore, has several limits, i.e., the imbalance of chemotherapy regimens administered. Furthermore, main analyses were conducted within the modified intention-to-treat population, in which every patient receiving at least one chemotherapy dose was included: Data show a heterogeneity of treatment compliance between both regimens with a high proportion of incomplete cycles in the 6-months group (independent of chosen regimen). A more consistent analysis including patients with complete adjuvant therapy (3 vs. 6 months) would be appreciated (per protocol analysis).

Considering the clinical point of view, absolute difference of 0.4% in OS rate at 5 years and, simultaneously, a significant improvement of PSN at 3 years should be included in the decision-making of adjuvant treatment. Thus, individual evaluation of adjuvant therapy including patient preference, relevant comorbidities, and other prognostic factors (i.e., tumor-related) is recommended.

#### 4.4.2. IDEA: Shortening Treatment Duration Also in High Risk Stage II Disease?

Besides stage III colon cancer, post-hoc analyses were also done in stage II tumors with clinical *high-risk* characteristics, as (mostly) defined by the MOSAIC [17] trial (poorly differentiated tumors, insufficient nodal harvest, tumor perforation, obstruction, vascular/perineural infiltration). Those were included in four trials (SCOT, TOSCA, ACHIEVE-2, HORG). 3273 patients were included into this analysis, results of 5-yr DFS were similar to stage III disease: Non-inferiority of the shorter regimens (HR 1.18, 80% CI, 1.05 to 1.31) and of the FOLFOX subgroup (HR 1.42, 80% CI, 1.19 to 1.70) was not shown. However, the CAPOX regimen administered for 3 or 6 months was non-inferior (HR 1.02, 80% CI, 0.88– 1.17) [27]. However, it has to be considered that stage II disease requires an even more sensitive selection of indications for oxaliplatin: in MOSAIC, after a median follow-up of 9.5 years, 10-year overall survival among all included patients with stage II disease has been without any benefit (HR = 1.00, *p =* 0.98). However, in patients with *high-risk* stage II disease defined as *T4, tumor perforation*, or *fewer than 10 lymph nodes* examined, estimated 10-year overall survival was 75.4% vs 71.7% (*p =* 0.058), defining oxaliplatin as a standard here [13].

In this risk group, results are heterogenous: The trial with the largest portion, SCOT, has shown no relevant differences between 3 and 6 months regarding 3-yr DFS (HR 0.988, 95% CI, 0.746 to 1.31). Other trials also failed to show non-inferiority (HORG: HR 1.05, 95% CI, 0.68 to 1.63) [23], ACHIEVE-2 (HR 1.12, 95% CI 0.67 to 1.87) [33]. However, the Italian TOSCA trial has shown a superiority in relapse-free survival for the 6 months treatment duration (HR 1.41; 95% CI 1.05 to 1.89) [34] although the 5-yr DFS was similar [20].

Therefore, 3 months of CAPOX can be regarded as a standard of care in patients with *high-risk* stage II disease.

## 5. Best Use of Oxaliplatin with FP: What Have We Learned from IDEA?

As it has been shown in all analyses of all subgroups, treatment with oxaliplatin is associated with both, efficacy and toxicity. Treatment duration strongly correlates with the occurrence of cumulative neuropathy. The goal of the IDEA collaboration was to show that the limitation of oxaliplatin treatment duration was not necessarily associated with clinically relevant poorer outcome – and the message learned is that individual decision-making based on many factors, including patient’s characteristics and choice of treatment, mainly with respect of fluoropyrimidines as combination partner, are of utmost importance. Recommendations for adjuvant therapy in stage III colon cancer based on the results of the IDEA collaboration contain the use of reduced cumulative doses of oxaliplatin due to shorter treatment duration, especially in the *low-risk* group (T1-3 N1) using CAPOX regimen for 3 months. Nevertheless, a chemotherapy period of six months should be advised to patients assessed as *high-risk* (T4 and/or N2) or if FOLFOX is the preferred chemotherapy regimen. In stage II patients with clinical *high-risk* features which show a sustained benefit from oxaliplatin treatment (T4, fewer than 10 lymph nodes), also a 3 months CAPOX treatment should be seen as the treatment of choice. Further investigations are needed to better define those risk groups. In future, further data from these six trials are warranted to identify more, clinical and molecular predictors to determine those patients who would clearly benefit from either adjuvant modality.

## 6. Conclusions: Treatment Duration: Where Are We Today?

**Figure 2:**
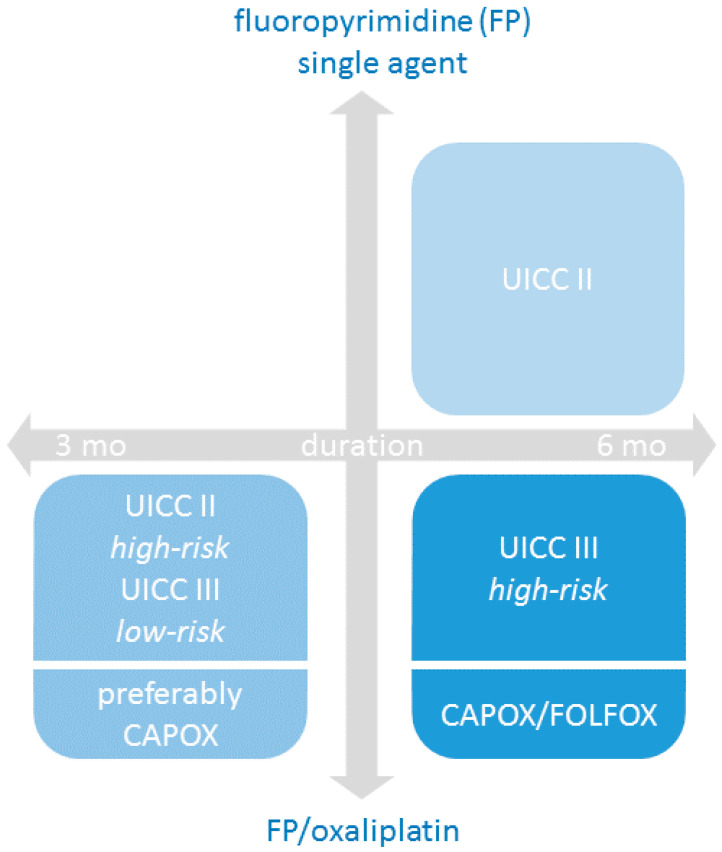
Recommendation for adjuvant chemotherapy in UICC II and III colon cancer depended on treatment duration, treatment intensity and clinical risk factors.

A uniform treatment duration of 6 months has been the standard of care with any regimen of FP or FP/Ox in stage II and III for more than a decade. Nowadays, the adapted approaches seem to be feasible and have replaced guideline-based treatment recommendation [35,36,37], given the absolutely seen small differences—with even statistically proven non-inferiority in some subgroups in stage III. With the proviso of conclusions from subgroup analyses from a large trial, key findings for stage III patients (Figure 2) are:

Oxaliplatin treatment duration can be shortened in patients with a (prognostic) *low-risk* features. Currently, this low risk is seen as pT1-3 with pN1.
In most prognostic groups, clinical differences are rather small—despite the fact that technically, the statistical non-inferiority of limiting oxaliplatin treatment duration has not been shown. Furthermore, the choice of FP/oxaliplatin regimen seems to be important. However, in all of those clinical risk/choice of FP subgroups (Table 1, yellow fields) the limitation of oxaliplatin treatment duration is individually to consider (Table 1, yellow fields).
A 6 months treatment duration seems to be mandatory in clinical *high-risk* patients if FOLFOX is the regimen of choice.

In stage II, results are less clear:

Six months of a FP alone remains a standard of care in patients with clinical *low-risk*-features, if a treatment indication exists and/or is considered at all.
In patients with *high-risk* stage II disease—with insufficient lymph nodes (<10), perforation and/or T4 stage—the indication for a combination treatment exists. The CAPOX regimen can be considered here with a treatment duration limited to 3 months (with some caution).
It remains unclear whether for an intermediate risk group (other risk factors than T4 and/or <12 lymph nodes), 3 months of FP/oxaliplatin could substitute a 6 months treatment with a FP as single agent.

Generally, like in selection of treatment at all (yes/no) and choice of treatment intensity (with/without oxalipatin), also treatment duration will need further refinement according to risk factors (prognostic) including new parameters, and predictive factors for gain of prognosis by shorter/longer duration. More parameters are under investigation. Next to the Immunoscore^®^, first analyses of ctDNA have shown that large prognostic differences can be detected here [29,31]. This prompted into the global Circulate IDEA collaboration [38]. Also, predictive factors for toxicity may play a role.

Furthermore, the physician’s experience is a valuable tool for choosing and administering adjuvant chemotherapy. Next to the risk assessment and treatment selection at ‘baseline’, a continuous assessment during treatment and its adaptation is of utmost importance: Absolute differences in efficacy are mostly rather small, compared to the immense differences in clinically relevant (cumulative) toxicity. Therefore, also a critical and careful patient´s toxicity assessment and modification throughout the treatment period are crucial.

However, the book of evaluation for optimal treatment durations is not closed—and not limited to treatment shortening. Upcoming therapeutic principles in adjuvant treatment will also question the optimal treatment duration. Specifically, immune checkpoint inhibitors have entered the arena: For patients with microsatellite instable (MSI high) and/or deficient mismatch repair system, ongoing trials are integrating immune checkpoint inhibitors, and as an example, treatment duration with these compounds are 12 months (additional six months after completion of cytotoxic chemotherapy), but without any evidence of the optimal duration in the immunomodulatory setting here [39].

## Figures and Tables

**Table 1:** yr DFS rate by risk group and regimen (modified by Grothey et al. [18] and Iveson et al. [27]).

			**Regimen**						**All-Patient Cohort**		
			**CAPOX**			**FOLFOX**					
			**3 yr DFS (%)** **(95% CI)**		**HR** **(95% CI)**	**3 yr DFS (%)** **(95% CI)**		**HR** **(95% CI)**	**3 yr DFS (%)** **(95% CI)**		**HR** **(95% CI)**
			**3 m**	**6 m**		**3 m**	**6 m**		**3 m**	**6 m**	
**UICC III**	**Risk Group**	**low-risk** **(T1–3 N1)**	**85.0** **(83.1–86.9)**	**83.1** **(81.1–85.2)**	0.85 (0.71–1.01)	81.9 (80.2–83.6)	83.5 (81.9–85.1)	1.10 (0.96–1.26)	83.1 (81.8–84.4)	83.3 (82.1–84.6)	1.01 (0.9–1.12)
		high-risk (T4 and/or N2)	64.1 (61.3–67.1)	64.0 (61.2–67.0)	1.02 (0.89–1.17)	61.5 (58.9–64.1)	64.7 (62.2–67.3)	1.20 (1.07–1.35)	62.7 (60.8–64.4)	64.4 (62.6–66.4)	1.12 (1.03–1.23)
			**Regimen**						**All-Patient Cohort**		
			**CAPOX**			**FOLFOX**					
			**5 yr OS (%)** **(95% CI)**		**HR** **(95% CI)**	**5 yr OS (%)** **(95% CI)**		**HR** **(95% CI)**	**5 yr OS (%)** **(95% CI)**		**HR** **(95% CI)**
			**3 m**	**6 m**		**3 m**	**6 m**		**3 m**	**6 m**	
**UICC III**	**Risk Group**	low-risk (T1–3 N1)	90.4 (88.9–92.0)	88.1 (86.3–89.8)	0.85 (0.69–1.04)	89.1 (87.8–90.5)	89.4 (88.1–90.7)	1.02 (0.87–1.19)	89.6 (88.6–90.7)	88.9 (87.8–90.0)	0.95 (0.84–1.08)
		high-risk (T4 and/or N2)	71.4 (68.7–74.2)	72.4 (69.7–75.2)	1.03 (0.89–1.20)	72.5 (70.2–74.9)	75.3 (73.1–77.6)	1.12 (0.98–1.27)	72.0 (70.3–73.8)	74.1 (72.4–75.9)	1.08 (0.98–1.19)
			**Regimen**						**All-Patient Cohort**		
			**CAPOX**			**FOLFOX**					
			**5 yr DFS (%)** **(80% CI)**		**HR** **(80% CI)**	**5 yr DFS (%)** **(80% CI)**		**HR** **(80% CI)**	**5 yr DFS (%)** **(80% CI)**		**HR** **(80 % CI)**
			**3 m**	**6 m**		**3 m**	**6 m**		**3 m**	**6 m**	
**UICC II**	**Risk Group**	high-risk	81.7 (n/a)	82.0 (n/a)	1.02 (0.88–1.17)	79.2 (n/a)	86.5 (n/a)	1.42 (1.19–1.70)	80.7	83.9	1.18 (1.05–1.31)

Green—non-inferior, yellow—not proven, red—inferior.

## Abbreviations

FP	Fluoropyrimidine
5-FU	5-Fluorouracil
IS	Immunoscore^®^
ctDNA	Cell-free tumor DNA
LDLV 5-FU	Low-dose LV
HDLV 5-FU	High-dose LV
LV	Leucovorin
LEV	Levasimole
NI	Non-inferiority
PSN	Peripheral sensory neuropathy
UFT/FA	Tegafur/folinic acid
